# Understanding youths’ concerns about climate change: a binational qualitative study of ecological burden and resilience

**DOI:** 10.1186/s13034-022-00551-1

**Published:** 2022-12-31

**Authors:** Isaiah Thomas, Andrés Martin, Antoine Wicker, Laelia Benoit

**Affiliations:** 1 Yale School of Medicine, Yale University, New Haven, CT USA; 2 Xavier Bichat School of Medicine, University of Paris VII, Paris, France; 3 CESP, Team DevPsy, Inserm 1178, Maison de Solenn, Hôpital Cochin AP-HP, Université Paris-Saclay, UVSQ, Paris, France

## Abstract

**Background:**

Climate change has been shown to have long-term effects on mental health, yet, to date, there have been few studies on how children and adolescents experience and respond to ecological changes and how and why they engage in climate action. We explored empirically young people’s views about climate change and how distinct cultural contexts influence individual climate action.

**Methods:**

We invited children and adolescents (ages 7 to 18) and their caregivers from the general population in the United States and France to participate in semi-structured focus groups. We recruited 74 participants, 39 in the U.S. (33 children and adolescents, 6 parents) and 35 in France (32 children and adolescents, 3 parents). Focus groups with participants centered on their emotions, beliefs, and actions around climate change. We analyzed the focus group data and developed themes via grounded theory and symbolic interactionist approaches.

**Results:**

Many participants described experiencing anger, hopelessness, guilt, and sadness in response to climate change, and a smaller number endorsed significant anxiety symptoms; many described frustration about needing to fix the mistakes of earlier generations. Younger participants frequently misunderstood the purpose of their parents’ eco-conscious behaviors unless they were provided with age-appropriate explanations. Participants described a spectrum of experiences when trying to discuss climate change with peers and family, ranging from genuine support to apathy to hostility. Between the two samples, U.S. participants experienced more conflict with adults about climate change than French participants, but French participants described a greater lack of political agency compared to U.S. participants. Participants in both samples expressed a relatively balanced view of climate action, recognizing the significance of individual actions while acknowledging the limits of their power in the face of systemic issues. Some found hope and empowerment through climate action and building communities around it.

**Conclusion:**

Discussing with children and adolescents what adults are doing to mitigate climate change can provide reassurance, model prosocial behaviors, and inspire their own investment in climate action. Adults seeking to support the psychological well-being of young people should both support their concerns and actions around climate change and create avenues for young people to meaningfully engage in climate action.

**Supplementary Information:**

The online version contains supplementary material available at 10.1186/s13034-022-00551-1.

## Introduction

Climate change has adverse effects on mental health, ranging from recurring worries to depression, panic attacks, and increased risk of suicide [1]. The term “eco-anxiety,“ or climate change anxiety, defined by the American Psychological Association in 2017 as “a chronic fear of environmental doom,“ has emerged in the climate psychology discourse as an umbrella term for the psychological symptoms associated with the climate crisis [2]. Eco-anxiety is not a psychiatric diagnosis according to the Diagnostic and Statistical Manual of Mental Disorders, Fifth Edition (DSM-5). Additionally, few studies thus far have investigated youths’ experiences with eco-anxiety, and no standardized criteria or scale currently exists for it in this population, despite children and adolescents being “among those who are most acutely experiencing the mental health impacts of climate change” [3]. A 2021 survey of 10,000 young people (aged 16–25 years) around the world found that most participants regard governments as failing to take climate action “in a coherent, urgent way” and experience feelings of betrayal and abandonment, “not just of the individual but of young people and future generations generally” [4].

We previously explored the narratives in U.S. newspapers about young people and climate change [5]. We found that these narratives cast young people in various archetypal roles: innocent victims, powerful activists, young saviors, or brainwashed zealots. Adult narratives tended to focus on the parents of young children, who felt reluctant to talk to their children about climate change for fear of making them anxious, and on the parents of adolescents, who viewed their teenagers’ actions with ambivalence.

Young people are rarely given the opportunity to speak for themselves about their views and emotions regarding climate change in conversation with their peers in a nonjudgmental space. Similarly, there are no published qualitative studies [6] exploring young people’s feelings and experiences about climate change. This is an important gap in the literature, given that younger generations are likely responding to the climate crisis differently in light of more widespread acknowledgment of climate change in school curricula, news media, and social media. Survey-based studies allow for only a limited range of responses and have largely focused on participants over the age of 15.

In this study, we aimed to allow children and adolescents of a wider range of ages to express their thoughts and feelings about climate change. Further, we sought to understand how their perspectives evolve in a developmental context. We chose to include participants from two high-income Western countries to better understand how cultural and political contexts shape youths’ understanding of climate change. Our goal was to identify actionable ways for parents, educators, clinicians, researchers, and policymakers to support youth in the context of climate change.

## Methods

We recruited participants from the general public—that is, not a clinical population—via advertisements shared on social media platforms used by young people (Instagram and TikTok). We requested input from the first group of participants on the imagery and language used in our advertisement, leading to its final version: “Child and Adolescent Earth Emotions. Looking for participants for a research study. Goal is to investigate child and adolescent behavior about climate change. Methodology is a one-hour focus group. Zero requirement, expectations, or criteria.” To enable in-depth conversations and reduce the risk of a confidentiality breach, focus groups were intended to be composed of four participants. Additionally, some participants were recruited via purposive sampling to ensure representation and diversity in regards to age, gender, race, education level, and socioeconomic status. To do so, we explained to participants the importance of representation in research and asked them to invite their peers with characteristics that were underrepresented in our sample. Participation in the study was voluntary, with compensation in the form of a $15/€15 gift card. We obtained informed assent/consent for participation and audio-recording of the focus groups from the participants and their parents. Inclusion criteria were ages 7 to 18 and proficiency in spoken English or French. Participants were asked to self-report their racial identity, level of education, and parents’ occupations, the last of which was used as a proxy for socioeconomic status.

We conducted focus groups stratified by age, each one lasting 60 minutes and conducted via synchronized videoconferencing. Two researchers (LB and IT) conducted the focus groups in English together, and one researcher (LB) led the focus groups in French. We elected to utilize semi-structured focus groups to allow participants to provide more spontaneous and detailed responses. The interview guide included sensitizing questions about their emotions surrounding climate change, their understanding of and beliefs about climate change, actions taken to address climate change, and various barriers and supports encountered when engaging in climate action [See Additional file 1 (Appendix 1)]. Focus groups with a small number of parents of younger participants (under 10 years old) helped us to better understand how their views and actions aligned with the perceptions of their children. We recorded, anonymized, and transcribed all focus groups.

Three researchers (IT, LB, AW) independently analyzed the data using grounded theory and symbolic interactionist approaches. First theorized by sociologists Glaser and Strauss in 1967, grounded theory is an inductive research methodology that attempts to generate theoretical frameworks that are “grounded” in data that has been systematically collected and analyzed [7]. As an epistemological framework of grounded theory, symbolic interactionism connects social structures with individual-level processes to better understand how individuals interact with one another to create symbolic worlds and how these worlds shape individual behaviors [8]. We argue that focus groups (in contrast to individual interviews) can reveal shared beliefs, identities, and collective knowledge, which are particularly meaningful when assessing underlying social relationships through symbolic interactionism. We wanted to not only understand participants’ personal experiences but how these experiences reflect and shape the participants’ social worlds, from family dynamics to peer interactions to participation in political systems.

We first analyzed transcripts in their original language, French (AW, LB), and English (IT, LB). Then, all codes and focus group transcripts were translated into English. The codes were discussed within the research group (IT, LB, AW, AM). In keeping with grounded theory methodology, we updated the interview guide iteratively to include additional questions that arose from the focus groups. The results were compared between focus groups to identify recurring themes, integrate new elements, and ensure triangulation and data sufficiency, that is, the point at which additional focus groups only supported identified themes and did not provide new themes or insights. Once sufficiency was achieved, we constructed a complete thematic description of the experiences of the participants, organized into overarching domains linked to underlying themes, each illustrated through verbatim quotations from the focus groups.

Given the central role researchers play in making inferences from their qualitative data, their positionality should be explicit. The principal investigator (LB) has expertise in qualitative research using sociological interactionism, an appropriate framework to study climate change emotions and actions in children. All researchers (LB, IT, AM, and AW) are child and adolescent psychiatrists (either in training or experienced), have experience in qualitative research, and have an interest in the impact of social injustice and cross-cultural challenges on children’s mental health.

## Results

Over the course of 2021 and 2022, we conducted 18 semi-structured focus groups with 39 participants in the U.S (33 children and adolescents, 6 parents), and 35 in France (32 and 3, respectively). As some participants had to reschedule their participation, focus groups included between two and four participants (mean = 3). Demographic characteristics of youth participants are listed in Additional file 2 (Appendix 2). Through iterative coding, we developed the three-domain model outlined in the following sections: (I) *Concerns about climate change*; (II) *Barriers to climate action*^2^; and (III) *Support for climate action*.^4^ We designate each verbatim quotation using the same convention throughout: age/gender.country (e.g., 15 F.Fr: 15-year-old French female; PF.US: female American parent).

### I. Concerns about climate change

When asked first about their understanding of climate change, all participants defined it along three dimensions: a warmer world, driven by human activities and resulting in environmental damage. Older participants detailed the mechanism leading to rising temperatures: burning fossil fuels result in greenhouse gas emissions, which trap the sun’s heat.

#### Climate emotions

When asked about the emotions that climate change evokes, answers such as, “angry,” “frustrated,” “sad,” “hopeless,“ and “guilty” were the most common. The anger and frustration were often in response to the apathy and inaction on the part of adults and previous generations. One participant described frustration with “people who kind of deny any individual responsibility because they feel like, ‘Oh, it’s beyond my control’” (18 F.Fr). A younger participant described feeling “disappointed in all the humans that are doing this” and wanting them to “change and just bring back the Earth’s regular climate” (7 F.US). Participants tended to experience guilt in terms of not doing enough to combat climate change: “I get sad because I like animals and also maybe guilty because I haven’t really done a lot of stuff to prevent it” (12 F.US).

Participants shared a range of responses regarding their feelings of anxiety, from no anxiety to occasional bouts of worry to lost sleep and low mood. Participants who viewed themselves as activists appeared more likely to describe symptoms of anxiety, often following a turning point in which they became acutely aware of the climate crisis: “When I was in middle school, I would feel this sense of dread because I used to think if I don’t go vegan, I’m going to die in a few years because the earth is going to combust” (16 F.US). Some identified certain situations as triggering anxiety about climate change: “When you have a lot of information at the same time about, basically, the planet is dying, it’s your fault […] We have a lot more information about what we did wrong than what we did right” (17 F.Fr).

Several participants had a manageable level of anxiety about climate change. One participant described himself as neither “paralyzed” by climate change anxiety nor “ignorant to the scale of the issue,” instead trying to “channel a lot of that anxiety into action” (15 M.US). Physical and social distance mitigated anxiety related to climate change for some: “I’ve never developed any anxiety about climate change because I don’t think I’m facing it at the moment” (17 F.Fr). One participant wondered if the urgency of climate change might itself be overblown: “A lot of times, the media and people, like politicians, will exaggerate the stuff. There’s figures that have been around for years and years and that are just not very correct” (16 M.US). Some described a level of ambivalence about the situation: “On the one hand, it’s scary because if we go on like this, we don’t know how it will end. On the other hand, I’m a bit hopeful because things have changed a bit in 10 years. So it’s ambiguous” (16 M.Fr) (Table 1).

**Table 1:** Domain I. Youths’ apprehensions about climate change

Theme	Sample quotation
Climate emotions	[Food disposal] causes me anxiety, because I feel like I have this huge responsibility on my shoulders. That when I throw things away, it’s wrong, it’s going to end up in the waste disposal center. I’m participating… in climate change. And I feel quite guilty (15 F.Fr).
Exposure to climate change information	*Through family:* She knows there’s droughts sometimes in California, especially in the past few years, and wildfires, and she will ask if it’s due to global warming, then we’ll talk about what causes global warming (PF.US). *Through media and technology:* Whenever I get nervous about something, I have my phone in my bed and I’m always looking stuff up. I definitely have lost some nights of sleep (17 M.US).
Younger children’s perceptions	One time I saw a video about polar bears not having homes. So I looked into that a little bit, but not that much (12 F.US).
Inheriting the problems of previous generations	They’re throwing the responsibility this way. It’s not something you throw at another generation. It’s something you’re supposed to share (17 M.US).
Identities and communities	A lot of people who are vegan, I feel like obviously they are doing it for the environment to a certain extent, but there’s also very much a culture of weaponizing that against other people and be like, “Oh my god, you’re not as cool as me” (17 F.US).
Green marketing	They really aren’t [sustainable]. And they’re really just trying to get a quick buck out of people’s collective anxieties over a serious issue (17 M.US).
Family planning	If we have children, I don’t want them to live in a world that is a garbage can, where you can’t breathe (12 F.Fr).

#### Exposure to climate change information

Older participants generally described their first exposure as occurring at school, usually in elementary or middle school. Few of them reported exposure to climate change information through their families: “The culture I grew up in was not unaware, but it wasn’t a part of our discussions” (15 M.US). However, some also felt that the topic was not covered sufficiently in school curricula: “It seems to me that it’s like sex education, there should be a number of hours of awareness, and that’s not done. [Both] are borderline taboo” (16 M.Fr). As a result, some participants had sought out information because of their own interest and curiosity and were discerning about their sources of information: “I think I did pretty well, to sort out the reliable sources [from YouTube], the not so reliable sources” (15 M.Fr). Notably, one older participant described minimal exposure to climate change information prior to the focus group: “I didn’t really have a good grasp of what climate change is before this” (17 M.US).

Younger participants more often reported first encountering climate change information via online content, at times increasing their fears and at others assuaging them. One parent described exercising caution when tackling the topic of climate change with her young children: “We haven’t ever discussed with them the severity of climate change. We don’t want to freak them out too bad” (PF.US). Another parent described using online media to teach her daughter about climate change: “[My daughter] was wondering what causes global warming. So, I showed her a couple videos. and she was interested in that” (PF.US). Another parent described her child seeking out climate change information online in preparation for participating in the study: “[My daughter] watched a few videos, and her level of expertise on the exact mechanism increased considerably. She felt like her role as an expert was explaining the environment to you seriously” (PF.US). Although this may reflect the impact of this research study on participants’ interest in climate change, rather than a spontaneous interest, it also suggests how younger participants can readily seek out information when they are curious about a topic.

#### Younger children’s understanding of climate change and climate action as concrete and immediate

Younger participants conceptualized climate change in more concrete ways as part of the changing environment, such as melting ice caps, rising sea levels, wildfires, and the loss of animal species, such as polar bears: “One time I cried about it because I was actually really upset. Because of all the photos I saw. Of the polar bears” (11 F.US). In terms of how human actions impact the environment, younger participants had varying levels of understanding:

“It’s caused by burning fossil fuels, like coal and oil” (10F.US).

“Does taking a boat also pollute?” (10F.Fr)

“If there are 30 people, [a train] pollutes much less because it carries just the weight of the tram, plus a few people. But if everyone has a small car, it pollutes more, and there is a lot more weight” (11M.Fr).

“Right now, I couldn’t say what I know or what I don’t know” (12F.Fr).

When they were concerned about the issue, some younger participants expected moral clarity and identified hypocrisy among some adults: “There are people on Earth trying to do their best. But there are others who don’t care at all” (11 M.Fr); “Owners of companies that burn fossil fuels, they kind of think that [climate change] is real, but they don’t want to say that because they won’t have a company if they don’t burn fossil fuels” (9 F.US).

Younger participants were most easily able to conceptualize climate action in terms of planting trees, trash cleanups, recycling, and conserving resources like water or electricity. However, these participants tended to understand these actions in terms of not being wasteful, typically because parents framed these concerns as such. The participants had not made the connection between these activities and climate change but were able to grasp the relationship when explained to them:

Participant: “I know my mom wants to become a vegetarian, but…”

Interviewer: “Oh. Is it because of climate change or for another reason?”

Participant: “I never asked” (9F.US).

“At home, we are more focused on food, that it has an impact on the climate and on the environment in general. It’s funny because it didn’t come up at all in the [focus group] discussions” (PF.Fr).

#### Inheriting the problems of previous generations

Many participants described frustration with previous generations, for both contributing to climate change and failing to act to stop it. Some attributed the inaction to denial and stubbornness: “They just didn’t care, they saw it as ‘This is my way of living, and I’ll be gone before it has any effect on anything’” (17 F.US). Another participant, who had taken a class on the history of environmental action, offered a more sympathetic explanation: “Really the earliest solid consensus came in the 70s. People did know, I guess is the factual answer. But, (a) they may not have necessarily known the gravity of the situation; and (b), these things take time” (17 M.US). Some described a sense of being abandoned by adults and left to fend for themselves: “I have the feeling that no one cares. They’ll say, ‘Anyway, I’m not the one who’s going to suffer, it’s going to be the children, other people’s children. It’s not my generation’” (17 M.Fr).

In contrast to the apathy and inaction of previous generations, some participants expressed greater hope about younger generations and climate action: “I don’t know if this is fair, but I feel like to a certain extent I’ve given up on older people. There’s people my age who care, our future, all that stuff” (17 F.US). One parent participant viewed climate change as an intergenerational problem requiring an intergenerational mindset to solve:

“I’ll say to [my daughter], ‘The planet belongs to you and to my grandchildren and it’s really important that we take care of it because we’re just borrowing it from the future” (PM.US).

#### Influences of cultural and socioeconomic factors on approaches to climate change

Among U.S. participants, white participants frequently denied an association between climate activism and racial and ethnic identities. Non-white participants were more likely to acknowledge the existence of stereotypes of climate activists as white, affluent, and inaccessible. One participant described how his cultural identity as Chinese-American complicated his engagement in climate action: “[My parents] were like, ‘How could you do this? You’re Chinese. You can’t be vegetarian. You can’t eat dumplings. You can’t eat all these different things’” (17 M.US). In spite of cultural associations between climate action and whiteness and affluence, participants described how these social groups were the least affected by climate change directly: “Climate change does disproportionately affect people of color, poor people, marginalized communities” (15 M.US); “So looking at Flint, Michigan, and a lot of other examples, [climate change] definitely unfairly impacts certain groups more than others” (18 M.US).

In spite of these challenges, a number of participants argued that the movement today is more accessible to a wider range of communities: “There’s a stereotype… but I feel like climate change is very open for everyone” (17 M.US). One parent participant who is an immigrant from China pushed back against the idea that being vegetarian is “not Chinese”: “It’s acceptable for me if [my children] chose to do that. Some of my friends in China, they are vegan” (PF.US). Thus, it appears that at least some portion of the conflict over climate action has been generational rather than cultural.

French participants emphasized cross-cultural barriers and differences rather than socioeconomic and ethnic divides; U.S. participants generally did not mention other countries when discussing social factors related to climate action. When discussing other countries, French participants tended to view them as either more or less advanced in comparison to themselves: “I have the impression that it’s much more accepted over there [in the United Kingdom] than in France where we are more into the ‘eat your meat’ traditions, that kind of thing” (15 F.Fr); “[In Serbia] there were attempts at climate protests, but they were directly stopped by the police. We were totally pacifist, but it didn’t work” (14 F.Fr). When issues around race and ethnicity came up, it was expressed with a level of distance: “It’s interesting to see the ‘ethnicity,’ and the whites and the blacks in the United States and the Black Lives Matter movement, and that’s what we haven’t done in France, maybe” (15 M.Fr).

Both French and U.S. participants mentioned how engaging in climate action can be challenging because of socioeconomic factors: “There is definitely a bit of a condescending nature to a lot of the people inside the vegan community. They’re not considering that a lot of people don’t have that option because of their financial situation, or a bunch of different factors” (17 F.US). Some participants feared that the socioeconomic factors tied to certain lifestyle changes could create more stigma: “There are those who do it, and then it creates discrimination against those who don’t. So, from a social point of view, it’s not great either” (15 M.Fr).

#### Growing skepticism about the intent and impact of green marketing

When asked about the marketing of products as “eco-friendly” or “green,” many participants expressed suspicion regarding the intentions and impact of such tactics. Some believed that brands would make small concessions in order to distract from larger issues: “It’s like, congrats, I’m drinking without a straw, but this cup of coffee was flown in from Costa Rica” (17 M.US); “I know they’re just using it to […] cover their ass and get people to think that they’re super socially conscious” (17 F.US). A few participants expressed a preference for companies with truly environmentally friendly values and practices; however, due to doubts about brands’ honesty, this type of marketing appeared to have relatively little impact on their purchasing practices.

#### Climate change triggering ambivalence around family planning

A relatively small number of older participants had heard of people planning to have fewer or no children in response to climate change; French participants appeared to be more familiar with the concept than U.S. participants. The question of having children, for those who had considered it, was “a scary what-if,” as one participant put it (17 M.US). One participant reported that climate change would be a major factor in such a decision: “Will we be around in a good enough state for that in the next 10 to 20 years? Because there is talk of it being irreversible pretty soon, so that’s definitely in my mind” (17 F.US). Some participants predicted a bleak future that dissuaded them from planning to have children: “Our own children will be even worse [off] than us and so on” (15 M.Fr). Some participants viewed adoption as a suitable alternative path to parenthood: “I will adopt because [the child] will already be born” (16 M.Fr).

Some participants said that in spite of their concerns, they plan to have children, albeit with climate change in mind: “I don’t know if I would compromise. Maybe I would have fewer kids than, say, my parents did” (18 F.US); “I want my two kids. Let’s start there” (17 F.US). Some participants felt that giving up having children for ecological reasons was unfair to future children: “It’s true that it’s going to help things, but I think it’s not being done for the children. There won’t be many of us left on this Earth” (14 F.Fr). One participant felt that the premise of limiting children for ecological reasons was itself flawed: “I very much disagree with restricting the amount of children. That’s a human right. It’s outlined in the Universal Declaration of Human Rights” (15 M.US). Another participant felt that we should focus instead on other more salient sources of carbon emissions: “We saw in geography class that having fewer children was not a solution, but rather reducing the carbon footprint of having children. It is more ‘ecological’ to never take a plane than to not have a child” (16 M.Fr).

### II. Barriers to climate action

#### Barriers to engaging in climate action oneself

##### Lack of agency over one’s personal choices

At the individual level, participants described difficulties being on the same page as their parents regarding climate action. Some felt guilty for going against their parents’ wishes: “I would feel really guilty about having to refuse meals and having extra meat lying around that I just wasn’t going to eat” (18 F.US). Others described a lack of agency in terms of making decisions about their household and lifestyle: “It’s really hard to have control over the bigger things, especially energy use in your house. You can’t really choose to have solar panels when you’re our age” (18 M.US). Even when they had the autonomy to make their own choices, participants encountered structural issues that prevented taking action: “People are very reliant on their own personal vehicles because, at least here, we don’t have very good public transport. I think the city needs to be investing a lot more in public transport” (15 M.US). Of note, multiple participants who lived in Paris described being able to forgo car use almost entirely through public transportation and walking.

Several French participants described their choices around eating animal products as easily becoming a source of tension within their families: “I often get remarks, and so do my sisters, that it’s stupid, we’re really ‘very stupid’ to not consume meat or milk, for example” (17 F.Fr). Because their families insisted that they continue consuming meat, some participants described limiting their meat consumption and emphasized locally sourced foods rather than being completely vegetarian or vegan: “I eat meat once a week at the most, and that must be on the weekend” (17 F.Fr); “I try to buy as much as possible from local shops, which guarantee good quality and a nearby source” (16 M.Fr) (Table 2).

##### Lack of agency to make structural changes

A number of French participants specifically mentioned the concept of “eco-delegates,” which refers to students selected to bring the ecological concerns of the student body to the attention of school administrators; four participants in the study had been elected eco-delegates at some point. They described disappointment with the limitations of the position; they considered the title to be one only in name, giving young people the illusion that adults are listening to them: “When we were labeled as eco-delegates, I had hopes, saying to myself that we were going to do real actions, that we were going to be able to do things, and of course, that wasn’t really the case” (17 F.Fr). School administrators appeared uninterested in having a dialogue, leaving young people feeling excluded from decision-making processes: “The sports teacher planned the eco-delegates meetings at a time when [my classmate] and I really couldn’t attend” (17 F.Fr). Participants described feeling blocked by administrative red tape and by a hierarchical structure in their schools.

#### Barriers to engaging others in climate action

##### Engaging with peers

Some participants described climate change as rarely coming up in conversation with peers because it was not relatable. As one participant put it, “It’s not a great icebreaker” (17 M.US). Multiple participants wanted to talk to peers about climate change but feared turning them off from it by doing so: “I’m a bit afraid of being judged and categorized as an extremist or whatever” (17 F.Fr). Even when they found peers who were interested in climate action, some participants were frustrated with virtue signaling among peers: “Sometimes it feels a bit like a fad for other kids. And while I appreciate that I am able to have these conversations and I do feel like we make sporadic changes based on it, it’s never a consistent kind of support and that is a little bit tough” (18 F.US). According to some participants, social media exacerbates this kind of half-hearted investment: “It’s just signaling to all your followers, ‘Oh my god, I’m so socially aware’” (17 F.US). Some participants felt that social media had limitations as a tool for change: “Social media mostly doesn’t inspire action further than awareness” (15 M.US).

##### Engaging with adults

When attempting to engage adults in action, many participants described facing dismissive attitudes toward their efforts. Some adults denied the reality of climate change: “My parents don’t really believe in climate change. And I can’t really argue too much with them because neither of them went to college or had very high education” (17 M.US). Other adults were dismissive specifically because of the participants’ age: “There’s also a certain amount of pride and ego when a bunch of people younger than you are trying to tell you that the way that you lived your life is wrong. A lot of it comes from adults being obsessed with being in this position of authority and control” (17 F.US). One participant put it in starkly generational terms: “Our generation thankfully is starting to think more about the government’s role and corporations’ role inside of climate change. The generation above us, they’re focused on the individuals, they’re yelling at people, ‘Oh, use a metal straw. Do this, do that.’ And the generation above that, they just don’t care. So, I feel like we are progressing” (17 F.US).

French participants in particular noted a distrust in policymakers’ supposed commitment to climate action: “There is no real action taken by governments, who like to make nice speeches, but I find it hard to see concrete actions and their consequences” (15 F.Fr); “It is purely a political strategy, to make promises that are not kept” (17 F.Fr). One participant cited a study claiming that only 10% of French citizens said that they trust their politicians. Although French participants overall preferred not to engage in demonstrations to make their ecological demands heard, one participant reported taking part in climate change protests: “I started directly to protest, to try to change people’s minds, to try to change the world; so we made some noise, it reached other schools in Paris, and so on, and we started to demonstrate” (14 F.Fr).

### III. Support for climate action

**Table 2:** Domain II. Youths’ perceptions about climate action: barriers

Theme	Sample quotation
Engaging in action oneself	*Lack of agency: personal choices:* My parents were very against me going vegetarian. For a couple of weeks, they made me cook my own food because they were like, “If you’re not going to eat meat, you’re not eating” (17 M.US). *Lack of agency: structural barriers:* The very purpose of “eco-delegates” is that the students suggest ideas and projects that they wish to carry out, but it is above all the school that must approve them (17 F.Fr).
Engaging others in action	*With peers:* At the end of the conversation, there’s a very much awkward “Okay, now what do we do?” So I feel like because of that, a lot of people are disincentivized from even bringing it up, just because it can feel so hopeless (17 F.US). *With adults:* They’re like, “I didn’t know, I didn’t do anything wrong, and I don’t want to change that” (17 F.US).

#### Support from family

For many participants, guidance and advice from family members were central to their decision to make lifestyle changes. For participants living in families that emphasized climate change awareness, adopting eco-friendly actions was relatively straightforward. One participant described learning from and imitating the ecological practices of her mother: “I’m lucky because my family, especially my mother, studied environmental technology, so she was already quite knowledgeable” (15 F.Fr). In the face of parental disapproval, support from siblings was crucial for a few participants’ engagement in climate action.

Parent participants described trying to support their younger children when they wanted to make lifestyle changes: “She occasionally will say, ‘I don’t know if we should eat whatever.’ And I’m like, ‘That’s up to you. Whatever you want. You tell me and then I’ll get it and make it.’ I mean, I certainly wouldn’t let her not eat her vegetables, but if she decided she didn’t want to eat meat, we would just stop” (PM.US). One parent participant described doing everything possible to ensure that their household consumed healthy products and discussed these issues with her son: “We’ve been sensitive to it for a long time” (PF.Fr) (Table 3).

#### Support from friends and peers

U.S. participants described relationships with friends and peers as the most consistent and meaningful support for climate action. Friendships appeared to be important springboards to engaging in climate action. When asked how his friends might react if he chose to cut meat from his diet, one participant reported, “I 100% believe that they would be really supportive. I have a couple friends who are actually vegan and vegetarian, and I’m just like, ‘Okay, go for it. That’s great’” (17 M.US). Another participant described finding solidarity and reciprocity in connections with peers engaged in climate action: “A lot of the people I’m friends with are constantly sharing around different resources, and we’re like, ‘Oh, are you coming to the climate change meeting today? Oh no, I can’t, I’ll go to the next one. I’ll make a poster for it’” (17 F.US). In the context of the COVID-19 pandemic, when many of these relationships were forced to go virtual, some participants found support from online friends: “Social media helped me connect with people who actually care about this all over the globe. I feel relieved that, OK, I’m not alone.” (16 F.US).

French participants reported comparatively fewer instances of peer-based organizing. One participant cheered up her friends who, after being eco-delegates the previous year, had felt hopeless about bringing awareness to students and the administration: “This will change. I went into the classes at the beginning of the year and talked about the eco-delegates. So I’m hopeful that some students will get the point” (17 F.Fr). Some French participants used social media to discuss environmental problems and build long-standing friendships based on shared concerns: “We have discussions with [four friends’ names] in our WhatsApp group. That’s where we became friends” (17 F.Fr).

#### Hope and empowerment through collective action

Outside of individual changes taken in the name of climate change, U.S. participants described several activities related to collective climate action. This included participating in protests and rallies, organizing awareness-spreading campaigns, and being part of local climate change organizations. They described focusing on “bigger actions” such as “convincing adults to try to switch over to other forms of energy” (18 M.US) and mentioned the importance of “voting for the right people to be in power,” which “would also impact communities in the area” (17 M.US). A few U.S. participants were part of a local climate action organization, described as “completely grassroots” and “completely run by high schoolers” (17 F.US). One participant had “staged a protest four days ago in front of the city hall” with friends, as part of this organization (15 M.US). Another reported trying to “help put in legislation to change how much greenhouse gas emissions go into the atmosphere” (17 M.US). Even a few younger participants had taken part in collective action: “At our school before, there was a lot of trash on the playground or in the bushes nearby. So, my friends and I, we made the announcement to stop littering, and we also picked up the trash” (10 F.US). U.S. participants described positive feelings emerging from connections, within and outside the climate change movement, through reaching out and bringing others into the fold: “Having those conversations actually does bring me a lot of joy and really meeting people where they are, even though a lot of them are also feeling frustrated, to bond over that but also get over that and encourage individual action” (18 F.US).

For some participants, engaging in climate action became a way to sublimate their environmental anxieties into positive actions, which then resulted in positive emotions: “A lot of the stress makes me feel like I got to do something—not hopeful but like, empowers me, to go do something” (15 M.US). Other participants felt the challenge of climate action represented an opportunity for large-scale change: “It’s definitely frustrating, but it’s sort of exciting that we’ll essentially be in creative mode, and we can rebuild our world the way we see fit” (17 M.US); “There is fear, frustration, but it also opens possibilities for reconstruction. This kind of crisis also allows us to think about new ways of existing” (15 M.Fr). The response to the COVID-19 pandemic offered hope to some participants that such large-scale changes were possible: “We’ve seen the medical community pull itself up in essentially one year to come up with a vaccine, and I guess that speaks to science’s ability to push, if given the resources and the time” (18 M.US).

#### Intergenerational support

Despite their frustration with adults, most participants expressed a desire for support and understanding from them. Some participants wanted adults to empathize with young people’s fears about the ecological crisis: “If your child or someone younger than you is doing something about it, instead of criticizing them, you should be the support system. Ask them if they need any help, how they’re feeling about it” (16 F.US). Others desired more direct action and less defensiveness from adults: “Having some more openness and willingness to change habits, which also can help their own health sometimes” (18 F.US). Some participants suggested that adults discuss climate change with younger children and model eco-conscious behaviors for them: “If you have someone in your family that you see regularly, it has a much bigger impact than social media or even a friend. Someone who will go through things with you, who will also sort of corroborate your own feelings and your own beliefs about climate change” (17 M.US).

## Discussion

### Eco-anxiety: a helpful label?

**Table 3:** Domain III. Youths’ perceptions about climate action: supports

Theme	Sample quotation
From family	My siblings definitely influenced me a lot in terms of being environmentally conscious. School made me aware, but I don’t think I would have taken action if I didn’t have someone in there with me, like, “Okay. Let’s go vegetarian together” (17 M.US).
From peers	I actually joined that climate change group because one of my friends was in it, and she pretty much got our whole friend group to join the climate movement (17 M.US).
Hope and empowerment	Looking at other people [in the Climate Change Collective] and going like, “Oh OK, there’s a lot of us, we can do this, we can do something.” It’s really nice, it’s really inspirational, even (17 F.US).

Psychiatrists in our research team observed that participants in this study did not endorse symptoms of eco-anxiety or severe mental distress as a result of climate change. Some participants did endorse intermittent symptoms, such as occasional lost nights of sleep or situational anxiety when trying to make ethical choices.

The lay public and experts’ perspectives about eco-anxiety, as a psychological symptom tied to a historical context, mirror in many ways the views about nuclear anxiety (the fear of nuclear war) experienced by civilians in the West during the Cold War [9]. Researchers were particularly concerned with the effects of nuclear anxiety on the wellbeing and functioning of young people [10, 11]. Similarly, the label of “eco-anxiety” for young people’s well-founded concerns about climate change risks pathologizing an appropriate response to a significant stressor. Viewing their experiences and emotions broadly through the lens of eco-anxiety loses sight of the cause of the distress and frames the source of the problem as internal, rather than external, to the young person.

Participants in our study described how an emphasis on individual action could leave them feeling helpless and hopeless. Similarly, young people’s frustration about inaction toward climate change risks leading to a lack of trust in older generations [4]. Collective action, on the other hand, offered participants a sense of empowerment, community, and hope. Indeed, collective action may be more effective than individual action for decreasing psychological symptoms associated with climate change [12].

### Differing cultural contexts for personal autonomy and political agency

U.S. participants seemed to view themselves as having a greater agency to act as they believe is right compared to French participants, and they seemed to tolerate a greater degree of discordance between themselves and their families. To take the example of dietary choices, conflicts for French participants appeared to stem largely from a disruption of culinary traditions. However, French participants appeared less inclined to create conflicts with parents or adults generally, as they appeared to perceive the gap between themselves and older generations on climate change as smaller than young people in the U.S. In contrast, U.S. participants who described conflicts with their parents around dietary choices emphasized the defensiveness of many adults in response to being told what to do, which they perceived as an infringement upon their personal freedoms. As such, although U.S. participants tended to demonstrate more license to make their own choices in their households, they also expressed feeling more alone in their choices.

As for political engagement, U.S. participants tended to describe greater involvement in peer-based organizations and protests, whereas French participants described a lack of options and frustration with the existing options, such as being eco-delegates. French participants in our study tended to describe being offered nominal positions of responsibility by adults who still did not take them seriously. U.S. participants with direct political involvement described needing to take matters into their own hands and form their own coalitions. The relatively recent history of the Civil Rights Movement in the U.S. serves as a demonstration that advocacy and civil action could create lasting change. American political life has historically demanded active contribution to the community, with citizens volunteering and advocating for themselves and on behalf of others [13].

### Shifting cultural contexts for climate change and social justice

Beyond the nature of their interactions with adults around climate change, French and U.S. participants differed in how they conceptualize the intersection of identities and other social issues with ecological concerns. Though many factors could contribute to this observed difference, such as the emphasis on racial identities in U.S. politics versus the ban on large-scale collection of data on race and ethnicity in France, the history of environmental racism [14] and the ensuing environmental justice movement in the U.S. may offer insight into the American approach to climate action. Because France does not have the same overtly racialized history of environmental injustice, issues of identity for French participants seemed to focus more on national identity, particularly comparing themselves to other members of the European Union. U.S. participants, on the other hand, remained largely American-centric when considering issues of identity.

For our study, we sought participants of diverse racial and socioeconomic backgrounds to understand how these factors might shape perceptions of climate change. Concern about climate change and the level of climate change activism varied across and within these groups. When the association between climate action and whiteness and affluence arose from the discussions, participants, particularly those not part of those social groups, recognized the existence of the stereotype but argued that such associations were becoming less common. From a historical perspective, the argument that only privileged children can experience anxiety related to sociopolitical challenges is not new. Nuclear anxiety was also considered as a niche issue limited to the children of white, affluent parents [11]. Based on interviews with children from marginalized racial and socioeconomic backgrounds, child psychiatrist Robert Coles argued that children and parents from these groups don’t have the bandwidth to be anxious about the nuclear arms race because of more immediate concerns.

On the contrary, participants in our study described climate change as an issue of widespread concern that they have been exposed to through both school and social media, thus upturning the need for parents who share their concerns. Further, some participants described climate change as a part of larger networks of injustice, such as environmental racism and capitalism-driven exploitation. In contrast to Cole’s view, young people subject to systems of oppression may demonstrate the capacity to be invested in social and political causes even as they face more immediate challenges.

### Harm reduction and collective action

Multiple participants in our study described a perspective on climate action that we could summarize as “You can do something, but you can’t do everything” and “Doing anything is better than doing nothing.” Though not described explicitly in these terms, many participants appeared to have intuitively taken on this sort of approach, whether in their own actions to mitigate climate change or by having conversations with people who are less invested in climate action. They were willing, for example, to limit the number of children they have in the future but were not willing to abstain from having children altogether. In other words, participants were applying harm reduction principles to their own engagement in climate action.

Harm reduction refers to interventions aimed at reducing the negative effects of health behaviors without expectations of complete or permanent elimination of the behaviors [15]. Messaging emphasizing moderation rather than abstinence allows flexibility to engage in “modified risks” by allowing individuals a greater sense of autonomy over their health [16]. Similarly, harm reduction could be applied to climate action at the individual level. Crucially, collective action facilitates the efficacy of the harm reduction approach; if most people make some effort, the effect on climate change would be far greater than the impact of a much smaller group of individuals doing everything possible to mitigate their ecological impact. Further, such a model asks less of any given individual than an absolutist model, and thus is more likely to obtain buy-in from a larger group of individuals.

### Implications for parents, providers, and policymakers

Many parents fear that learning about climate change will engender anxiety in their children and that engaging in climate action will only exacerbate their fears. However, based on our findings, the more significant threat to young people’s mental health appears to be an awareness of the gravity of the climate crisis without a means of channeling these anxieties into action. One participant drew a comparison between sex education in schools and climate change education, with both being taboo. And as with sex education, children and adolescents are going to learn about climate change and its consequences, regardless of their parents’ interventions. Therefore, parents and providers may respond to young people’s concerns by initiating conversations and identifying avenues for meaningful engagement in climate action.

Regarding younger children, as perceptive and interested in climate change as these participants showed themselves to be in these focus groups, it was also clear that there were gaps in their understanding, particularly about what the adults in their lives were doing about climate change. Some parent participants were reluctant to discuss their eco-conscious habits because they did not want to scare their younger children. Nevertheless, explanations about the reasoning behind what adults are doing appeared essential to help children understand climate action. Thus, besides modeling pro-environmental behaviors, parents should communicate their intentions and embody a narrative of collective action. Such a *living narrative* can give young children a framework to understand climate action, a sense of security and being cared for, and an incentive for their participation in climate action.

### Limitations

We concede four main shortcomings. First, we included subjects from two high-income countries, making our findings potentially less relevant to low- and middle-income countries. Of note, another branch of our research study includes subjects from a middle-income country [17]. Second, the majority of participants in both samples were white and had parents in professional careers, and thus our findings likely under-represent the experiences and views of marginalized racial, ethnic, cultural, or socioeconomic groups in each country. Additionally, although we aimed to identify experiences that were shared among various demographic groups in each country, we recognize that the validity of cross-cultural generalization is limited, given the heterogeneous blend of cultures existing in each country. Third, our sample included a greater number of adolescents than children, and as such our findings disproportionately reflect the views of older and more cognitively and emotionally developed youth. Fourth, through focus groups, we may have introduced the opportunity for groupthink or participants responding in socially desirable ways, issues that individual interviews could have prevented.

## Conclusion

Our findings suggest that many young people are angry and frustrated, rather than anxious, about the climate crisis and the burden they face of fixing the mistakes of previous generations. Nevertheless, they seek help from older generations and do not believe they can do it all on their own. They are *adultified* children, trying to take on the responsibilities of adults, but they are still children at the end of the day, subject to the pressures of family and peers. Many do not identify as activists out to save the world but as part of networks of young people taking a thoughtful and measured approach to climate action. If our binational sample shared many commonalities, cultural differences between France and the United States shaped young people’s perceptions of their sense of agency, of the role of their governments, of the intersection between social and climate justice, and of families and schools as spaces either supportive or dismissive of grassroots climate action.

Although many adults fear that children learning about climate change and engaging in climate action will engender anxiety, based on our findings, the greater threat to young people’s mental health may be an awareness of the gravity of the climate crisis without a means of processing and redirecting these anxieties. Parents, researchers, and providers may respond to young people’s concerns by identifying avenues for meaningful engagement in climate activism [18]. Discussing with young children what adults are doing to combat climate change can provide reassurance, model prosocial behaviors, and inspire their own investment in climate action—and their generation’s.

## Supplementary Information

**Additional file 1.** Interview Guide.

**Additional file 2.** Demographic characteristics of U.S. and French youth participants (n = 69).

## Data Availability

All data generated or analyzed during this study are included in this published article and its supplementary information files.
