# Supplementary material for: Understanding youths’ concerns about climate change: a binational qualitative study of ecological burden and resilience
Source: Child Adolesc Psychiatry Ment Health. 2022 Dec 31;16:110. doi: 10.1186/s13034-022-00551-1 (PMC9805369; doi:10.1186/s13034-022-00551-1)
Supplement: Supplementary file 1 — Additional file 1. Interview Guide. [file 13034_2022_551_MOESM1_ESM.docx]

**Interview Guide**

**A. Sensitizing questions included in the initial version (March 2021)**

1. Are you **interested** in climate change, or not really? What does it mean to you?
2. What are your **emotions** regarding climate change (negative, positive, none)?
3. Have these emotions sometimes been so strong that you felt **paralyzed**?
4. What **raised your awareness** of climate change? An event? A discussion? Or was it gradual?
5. Have you ever **discussed** climate change with your friends/parents/other adults?
6. What are the possible **barriers to discussing** climate change with friends/parents/other adults?
7. Have you found interesting **information** on your own (on the Internet, social media)?
8. Have you **ever taken action** to reduce your carbon print, or not really?
9. **What are the actions** that you have already tried?
10. What are the **possible barriers to taking action** to reduce your carbon print? Example?
11. Have you ever taken part in **a discussion or shared activity that helped you** take action?
12. Can **peer-to-peer discussions** influence involvement in collective action?
13. How could **adults** be more helpful?

**B. New questions subsequently added (Last Update: December 2021)**

*In keeping with Grounded Theory methodology, successive iterations of the interview guide included new questions based on topics that arose during the focus groups.*

1. Do you think that climate change **has an impact on your mental health**, or not really? *[second version of question #3, more explicit]*
2. Do you feel that some companies are changing their **marketing** to appear eco-friendly, or not really? Does this make you more interested in buying their products? *[exploring green marketing, virtue signaling]*
3. Do you feel that your **individual actions can make an impact on** climate change, or not really? *[exploring individual responsibility, agency, and helplessness in face of structural challenges]*
4. Do you think that people of color, immigrant groups, or people in other countries experience climate change concerns in a specific way, or not really?
5. Some people have the opinion that climate change is a “**white people**” or “**rich people**” problem. Have you come across this view, or not really? What do you think about it?
6. Has the **Black Lives Matter** movement and protests in June 2020 influenced your concerns/behaviors around climate change, or not really?
7. Has the **COVID-19 pandemic** influenced your concerns/behaviors around climate change, or not really?
