# Supplementary material for: Understanding youths’ concerns about climate change: a binational qualitative study of ecological burden and resilience
Source: Child Adolesc Psychiatry Ment Health. 2022 Dec 31;16:110. doi: 10.1186/s13034-022-00551-1 (PMC9805369; doi:10.1186/s13034-022-00551-1)
Supplement: Supplementary file 2 — Additional file 2. Demographic characteristics of U.S. and French youth participants (n = 69). [file 13034_2022_551_MOESM2_ESM.docx]

**APPENDIX 2**

**Demographic characteristics of U.S. and French sample participants (n = 69)**

|  | **U.S.**  **n =39 (33 youth, 6 parents)** | **France**  **n=35 (32 youth, 3 parents)** |
| --- | --- | --- |
| **Youths’ age**  Years (%) | Ages 7-12: 15 (45)  Ages 13-18: 18 (55)  Median: 13 | Ages 7-12: 11 (34)  Ages 13-18: 21 (66)  Median: 14 |
| **Youths’ gender**  n (%) | Male: 13 (39)  Female: 20 (61) | Male: 14 (41)  Female: 18 (59) |
| **Youths’ race/ethnicity or origins**  (self-reported)  n (%) | White: 20 (61)  Asian*: 7 (21)  Black: 5 (15)  Latino/a: 1 (3) | Europe: 23 (72)  North Africa/Middle East: 9 (28) |

* Includes East Asian, South Asian, Southeast Asian, and Middle Eastern
